# Transcriptome-Wide Gene Expression in a Murine Model of Ventilator-Induced Lung Injury

**DOI:** 10.1155/2021/5535890

**Published:** 2021-04-07

**Authors:** Zhao Li, Guoshao Zhu, Chen Zhou, Hui Wang, Le Yu, Yunxin Xu, Li Xu, Qingxiu Wang

**Affiliations:** ^1^Department of Anesthesiology, East Hospital, Tongji University School of Medicine, Shanghai 200120, China; ^2^Department of Anesthesiology, Quanzhou First Hospital Affiliated to Fujian Medical University, Quanzhou 362000, China; ^3^Department of Anesthesiology, The First People's Hospital of Changde, Changde 415000, China

## Abstract

**Background:**

Mechanical ventilation could lead to ventilator-induced lung injury (VILI), but its underlying pathogenesis remains largely unknown. In this study, we aimed to determine the genes which were highly correlated with VILI as well as their expressions and interactions by analyzing the differentially expressed genes (DEGs) between the VILI samples and controls.

**Methods:**

GSE11434 was downloaded from the gene expression omnibus (GEO) database, and DEGs were identified with GEO2R. Gene Ontology (GO) and Kyoto Encyclopedia of Genes and Genomes (KEGG) pathway enrichment analyses were conducted using DAVID. Next, we used the STRING tool to construct protein-protein interaction (PPI) network of the DEGs. Then, the hub genes and related modules were identified with the Cytoscape plugins: cytoHubba and MCODE. qRT-PCR was further used to validate the results in the GSE11434 dataset. We also applied gene set enrichment analysis (GSEA) to discern the gene sets that had a significant difference between the VILI group and the control. Hub genes were also subjected to analyses by CyTargetLinker and NetworkAnalyst to predict associated miRNAs and transcription factors (TFs). Besides, we used CIBERSORT to detect the contributions of different types of immune cells in lung tissues of mice in the VILI group. By using DrugBank, small molecular compounds that could potentially interact with hub genes were identified.

**Results:**

A total of 141 DEGs between the VILI group and the control were identified in GSE11434. Then, seven hub genes were identified and were validated by using qRT-PCR. Those seven hub genes were largely enriched in TLR and JAK-STAT signaling pathways. GSEA showed that VILI-associated genes were also enriched in NOD, antigen presentation, and chemokine pathways. We predicted the miRNAs and TFs associated with hub genes and constructed miRNA-TF-gene regulatory network. An analysis with CIBERSORT showed that the proportion of M0 macrophages and activated mast cells was higher in the VILI group than in the control. Small molecules, like nadroparin and siltuximab, could act as potential drugs for VILI.

**Conclusion:**

In sum, a number of hub genes associated with VILI were identified and could provide novel insights into the pathogenesis of VILI and potential targets for its treatment.

## 1. Introduction

Mechanical ventilation is widely used in surgery and intensive care and offers a substantial advantage in managing patients' breathing during general anesthesia. It is also regarded as one of the most important means to treat patients who are undergoing respiratory failure as well as acute or chronic lung injury [1]. However, mechanical ventilation is a double-edged sword during respiratory support in some cases [2]. For example, improper use of mechanical ventilation may aggravate the original pathological damage or lead to direct lung injury, known as ventilator-induced lung injury (VILI) [3]. Patients with no original acute lung injury may develop acute lung injury after being subjected to mechanical ventilation for more than 48 hours [4]. Nearly half of the patients who had received mechanical ventilation for more than two weeks got pulmonary complications associated with mechanical ventilation. For this reason, it is of great pertinence to elucidate the pathogenesis of VILI and take protective or therapeutic measures against it. A lot of efforts have been made to reduce VILI occurrences during perioperative anesthesia and in critical care units.

At present, the primary measure taken for VILI prevention is the lung protective ventilation approach in which a small tidal volume is used, but its efficacy is highly limited [5]. The pathogenesis of VILI is still unclear, and effective interventions have yet to be investigated [6]. Therefore, it is vital to identify the involved key genes to better understand the molecular mechanism of VILI pathogenesis for an early, effective intervention.

As a powerful analytic tool, bioinformatics analysis has gradually been used in predicting the molecular mechanisms of VILI pathogenesis. For instance, Tamás et al. conducted the gene expression analysis in a mouse overventilation model and validated upregulated expressions of five genes (Areg, Akap12, Nur77, Cyr61, and Il11) [7]. Similarly, Ma et al. analyzed genes affected by VILI and validated the expression levels of randomly selected genes [8]. Despite those reports, the VILI-correlated hub genes have not yet been identified. In this study, analyses of hub genes that could be associated with VILI were performed based on extensive gene expression data of a mouse VILI model published online, to study their potential and specific roles in VILI.

## 2. Materials and Methods

### 2.1. Gene Chip Analysis and Identification of DEGs

**Figure 1:**
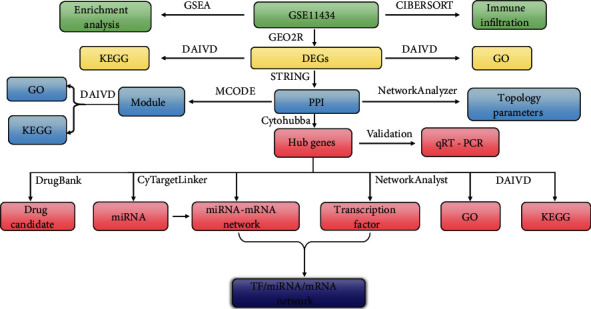
Flow chart of investigations in this study.

The procedures of analysis in this study can be seen in Figure 1. GEO is a database for storing and distributing the sequencing data [9]. In this study, we downloaded the GSE11434 dataset from GEO, based on the GPL1261 [10, 11]. GSE11434 was used to identify the hub genes associated with VILI. GSE11434 consisted of ten microarray chips and was divided into two groups: the VILI and control groups, with five samples in each group. The criteria of adj. *p* < 0.05 and ∣log_2_ FC | >1 were applied for screening DEGs.

### 2.2. GO and KEGG Enrichment Analysis

GO defines and standardizes terms for describing genes and their products, which contain three aspects [12]: cellular component (CC), molecular function (MF), and biological process (BP). CC is used to describe the area where the gene products are located in cells and could be a cellular substructure, an organelle (such as cytoplasm and nucleus), or a gene product set (such as a major histocompatibility complex). MF describes the function of gene products, such as carbohydrate binding and ATP-dependent hydrolase activity. BP specifies a more complex and advanced form of function systematically formed by a particular set of molecular process, such as mitosis and purine metabolism. DAVID (Ver. 6.8) was used to conduct the GO and KEGG enrichment analysis of DEGs [13]. The Benjamini-Hochberg corrected *p* value (*p* < 0.05) was set as the threshold for statistically significant enrichment.

### 2.3. Protein-Protein Interaction (PPI) Network Construction and Hub Gene Identification

PPI represents the process of forming a protein complex by noncovalent bonding between proteins [14]. The gene set data were imported into the STRING database to analyze their interaction. A confidence score ≥ 0.4 was considered as statistically significant. A PPI network graph was retrieved based on this standard.

Cytoscape (Ver. 3.7.1) is open-sourced software for bioinformatics analysis and is commonly used to visualize the molecular interaction network [15]. NetworkAnalyzer, a Cytoscape plugin, was used to perform topological analysis. The results exported from the STRING database were imported into Cytoscape to identify the top 10 DEGs by applying five algorithms in cytoHubba, and the overlapping genes of them were considered as hub genes [16]. The MCODE was used to screen out the statistically significant modules which were visualized by using Cytoscape [17].

### 2.4. Analysis of miRNA and TFs Related to VILI

CyTargetLinker is a plugin for Cytoscape that can extend the biological regulatory interactive network [18] and can be used to analyze the interactive relationship between various miRNA targets. In this study, we downloaded the murine gene datasets and selected miRTarBase, MicroCosm, and TargetScan databases to predict the regulatory relationship between hub genes and miRNAs. NetworkAnalyst was used to predict the TFs that could regulate VILI-associated genes [19]. Hub genes were selected in the above cases of prediction. The miRNA-TF-hub gene network was constructed using Cytoscape.

### 2.5. Gene Set Enrichment Analysis (GSEA)

GSEA uses a predefined gene set that can sort genes according to the degree of differential expression between samples from different groups and can subsequently validate whether the preset gene set is enriched at the top or bottom of the sorting list [20]. We downloaded the GSEA software from its official website and run it in a Java environment following instructions from a previous literature. Later, a curated KEGG gene set was downloaded from the MSigDB database, and then, an enrichment analysis was conducted according to the weighted enrichment statistic method in GSEA. The random number was set to 1000 to calculate the normalized enrichment score (NES) and false discovery rate (FDR). In GSEA, gene sets were considered significantly enriched when meeting the condition of NES≧1.0, NOM *p* < 0.05, and FDR≦0.25.

### 2.6. Immune Cell Composition Analysis

CIBERSORT is an algorithm that could be applied to estimate cell composition in complex tissues based on standardized gene expression data [21]. In this study, we used CIBERSORT to assess the relative proportion of 22 immune cells in each lung tissue. The mRNA expression profiling data of lung tissues from the VILI and control groups were extracted. Then, these data were calibrated using the Limma package in R. Subsequently, the LM22 signature matrix was applied in 1000 arrays to predict the proportion of immune cells. The samples were screened at the significance level of *p* < 0.05. The histogram of the proportion of each type of immune cell, the heat map of immune cell expression, the violin plots, and the correlation chart of immune cell proportion in lung tissues were plotted accordingly.

### 2.7. Identification of Potential Drugs

DrugBank is a database that integrates detailed data of drugs and comprehensive information of the drug target [22]. The identified hub genes were analyzed in the DrugBank database to determine potential molecules associated with VILI.

### 2.8. Animal Preparation and Experimental Protocol

Ten healthy specific pathogen-free male ICR mice (20-25 g) were procured from Shanghai JSJ-Lab and were separated in two groups: the VILI group (in which mice received mechanical ventilation) and the control group (in which mice breathed without any mechanical assistance). Mice were abstained from food 4 hours prior to anesthetization by intraperitoneal injection of 7.5% pentobarbital sodium solution (75 mg/kg). After the mice forming the VILI group were deeply anesthetized, they then received mechanical ventilation. Mice of the mechanical ventilation group were ventilated with tidal volume of 30 ml/kg, 65 breaths/min, and fraction of inspired oxygen of 0.21. Mice from the control group were allowed to breathe spontaneously. After 4 h of mechanical ventilation, the mice were euthanized, and then, lung tissues were harvested.

### 2.9. RNA Extraction and Quantitative Real-Time Reverse Transcription PCR (qRT-PCR)

The total RNA was extracted from mice lung tissues using TRIzol reagent (Takara, Japan). cDNA synthesis was performed using the PrimeScript RT Reagent kit (Takara, Japan). qRT-PCR was operated with the SYBR Green method (Qiagen, Germany) on the QuantStudio 7 flex real-time PCR system. The cDNA was used as templates to perform qRT-PCR for 40 cycles under the following conditions: an initial denaturation at 95°C for 20 s, followed by denaturation at 95°C for 15 seconds, and annealing at 60°C for 1 minute. The primers for cDNA sequences used in qRT-PCR are listed in Supplemental Table (available here). The mRNA expression levels of target gene were normalized to that the housekeeping gene GAPDH. The *ΔΔ*Ct method was used to calculate expression fold change of target genes.

### 2.10. Statistical Analysis

The histograms of gene expression were plotted using GraphPad (Ver. 8.0). The gene expressions were expressed in the form of mean ± standard deviation (SD). The comparison of differences between the VILI group and control was performed by independent samples *t*-test. *p* < 0.5 was deemed as denoting statistical significance.

## 3. Results

### 3.1. Identification and Enrichment of DEGs between the VILI and Control Groups

**Figure 2:**
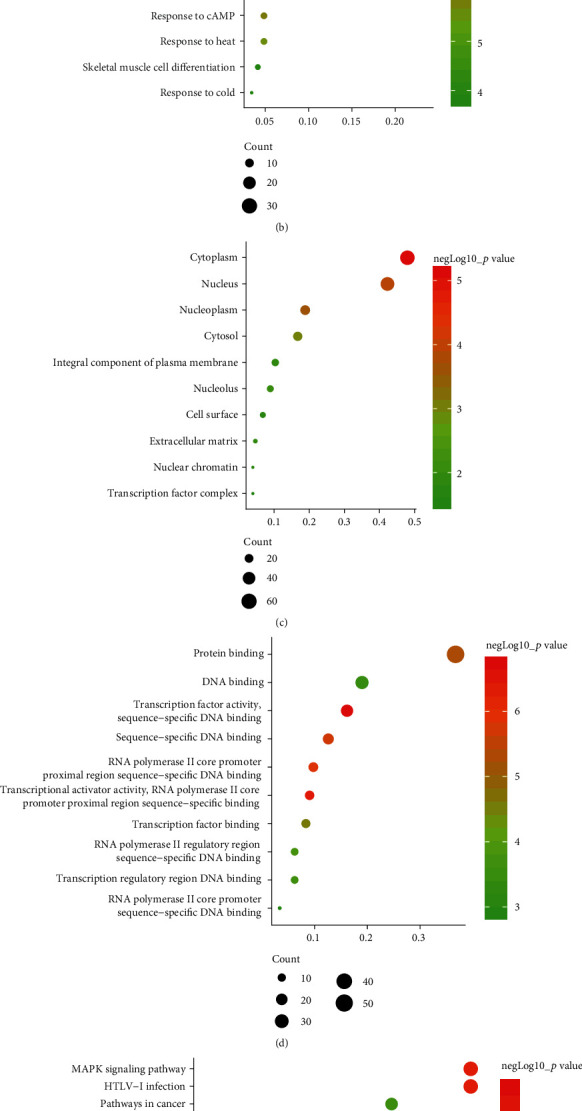
DEGs and enrichment analysis in the VILI and control groups. (a) Volcano plot of all DEGs in GSE11434. Red dots indicate upregulated genes and green dots indicate downregulated genes. (b) Top 15 terms in BP. (c) Top 15 terms in CC. (d) Top 15 terms in MF. (e) Top 15 KEGG pathway analysis.

To identify VILI-associated DEGs, we downloaded the GSE11434 expression profiles from GEO. A total of 141 DEGs were evaluated with GEO2R by following the criteria of adj. *p* < 0.05 and ∣log_2_ fold change | ≥1. Among them, 108 genes were upregulated and 33 were downregulated (Figure 2(a)).

To determine the specific functions of DEGs, GO annotation and KEGG pathway analyses were conducted with DAVID. In terms of BP, these DEGs were mainly involved in positive regulation of transcription from RNA polymerase II promoter as well as responses to lipopolysaccharide and cAMP (Figure 2(b)). In terms of CC, they were mostly enriched in the cytoplasm, nucleus, and nucleoplasm (Figure 2(c)). And primary enrichments in MF were mainly associated with TF activity, sequence-specific DNA binding, and protein binding (Figure 2(d)). *p* values were ranked in ascending order based on the results of pathway enrichment analysis. The top 20 enriched pathways, including MAPK, JAK-STAT, and TLR signaling, are shown in Figure 2(e).

### 3.2. PPI Network and Module Analysis of DEGs

**Figure 3:**
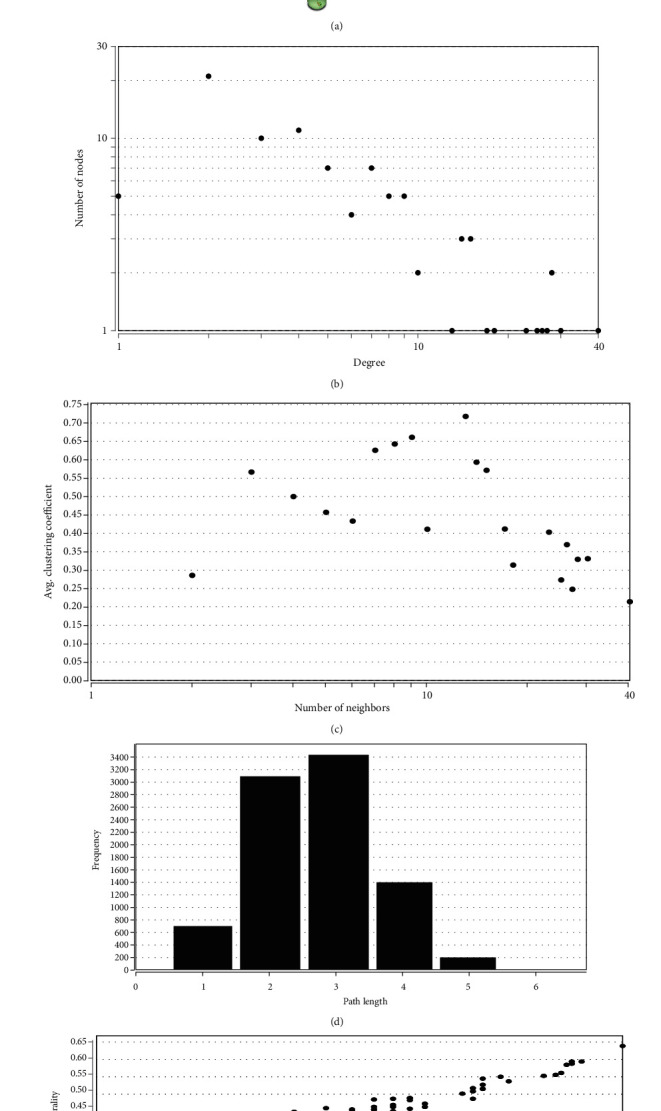
PPI network and module analysis. (a) PPI networks of DEGs. The topological parameters of PPI networks were as follows: (b) distribution of degrees, (c) clustering coefficient, (d) distribution of the shortest path, and (e) closeness centrality. (f) Most significant clustering module screened by MCODE from PPI networks.

To determine the interactive relationship between VILI-associated DEGs, an interaction network of DEG-encoded proteins was constructed. A total of 141 DEGs were mapped into the STRING database to retrieve a PPI network graph. As shown in Figure 3(a), the network included 133 nodes (target proteins) and three hundred and forty-eight edges (PPI). Topological analysis of the constructed PPI network was conducted using NetworkAnalyzer and revealed that the network topological parameters followed a power law distribution (Figures 3(b)–3(e)). The most significant module was screened out from the PPI network using MCODE, and 14 genes were identified (Figure 3(f)). We conducted functional enrichment analysis for these genes and found that they were mainly enriched in TNF and JAK-STAT signaling pathways (Tables 1 and 2).

### 3.3. The Identification and Validation of Hub Genes

**Figure 4:**
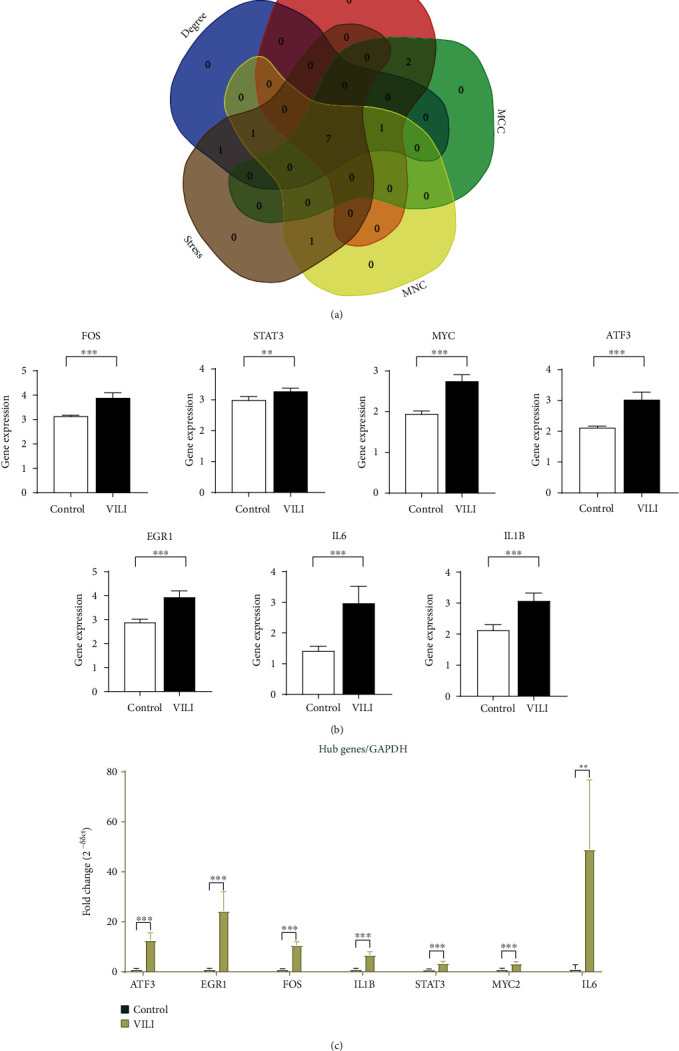
Hub genes in the VILI group. (a) Intersecting genes selected as hub genes by using 5 algorithms in cytoHubba. (b) The expression levels of seven hub genes in the lung samples from the VILI and control groups in GSE11434 were as follows: FOS, STAT3, MYC, ATF3, EGR1, IL-6, and IL-1B. (c) qRT-PCR analysis of hub genes in lung samples of the VILI and control groups.

To determine the VILI-associated hub genes, cytoHubba was used throughout the PPI network construction. Five algorithms (Degree, EPC, MCC, MNC, and Stress) present in cytoHubba were utilized to evaluate hub genes. The top 10 genes identified by each algorithm were intersected to obtain hub genes, which included Fos Proto-Oncogene (FOS), MYC Proto-Oncogene (MYC), Signal Transducer and Activator of Transcription 3 (STAT3), Early Growth Response 1 (EGR1), Activating Transcription Factor 3 (ATF3), Interleukin-6 (IL-6), and Interleukin-1 Beta (IL-1B) (Figure 4(a)). Notably, all those hub genes were contained in the most highly connected module mentioned above. In comparison with the control, the expression levels of all seven hub genes were upregulated in the VILI group (Figure 4(b)). We found out that the functions of these hub genes were mainly about inducing inflammation and TLR signaling pathway regulation. Then, we performed qRT-PCR experiment for further validation. The results showed that the hub genes were all overexpressed in VILI tissues, which is consistent with the prediction results (Figure 4(c)). GO and KEGG enrichment analyses of hub genes were then conducted. It turned out that hub genes were largely enriched in TLR and JAK-STAT inflammatory signaling pathways (Tables 3 and 4).

### 3.4. GSEA

**Figure 5:**
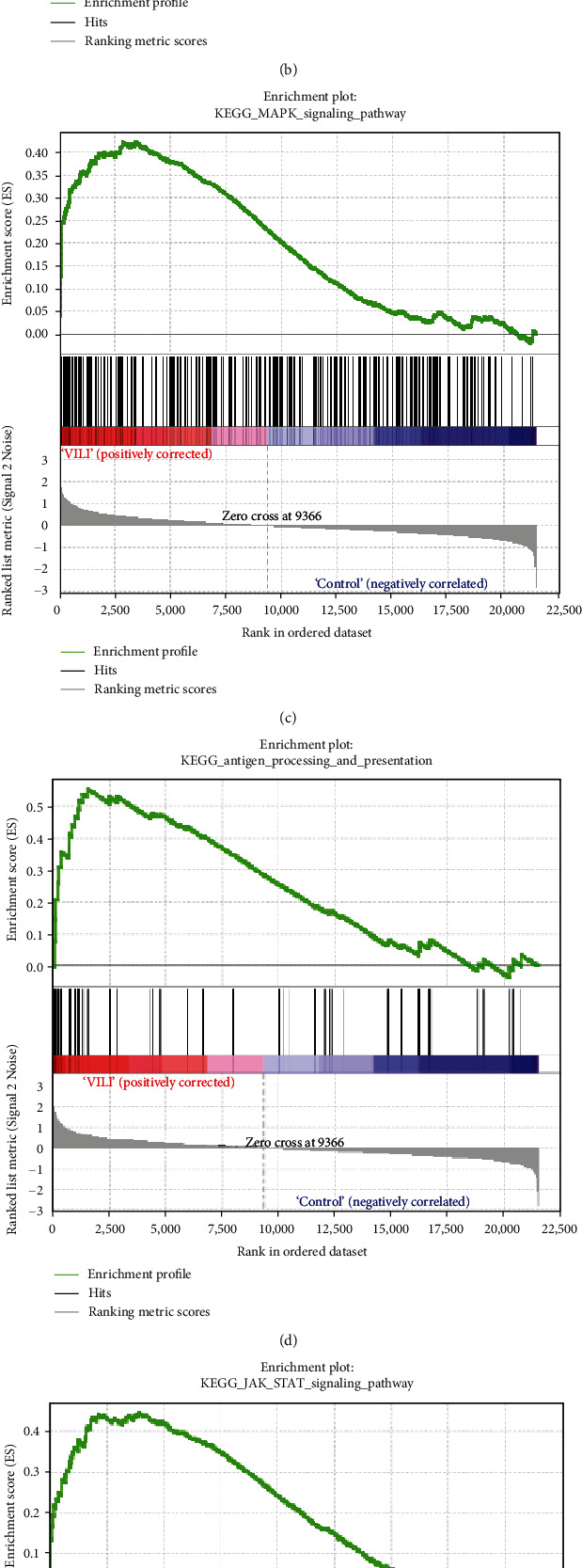
GSEA of six primary pathways in which VILI was significantly enriched.

GSEA was performed to determine the gene sets that had significant difference between the VILI and control groups. The gene sets positively correlated with the VILI group were mainly involved in the TLR and JAK-STAT pathways, which was consistent with the results obtained in GO and KEGG enrichment analysis (Figure 5). It was revealed that they were also enriched in NOD, antigen presentation, and chemokine pathways.

### 3.5. miRNA-TF-Hub Gene Regulatory Network

**Figure 6:**
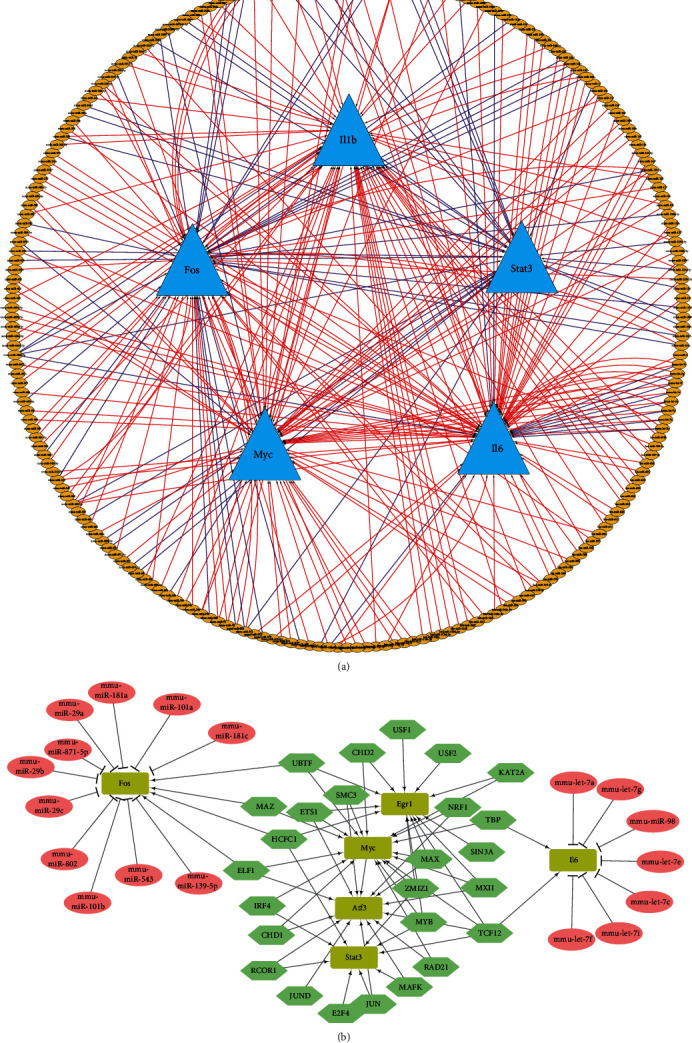
miRNA-TF-hub gene regulatory network. (a) miRNAs related to hub genes predicted by CyTargetLinker. Red edges mean predicted by MicroCosm and velvet edges mean predicted by TargetScan. (b) TFs related to the hub genes were predicted by NetworkAnalyst and then used to construct the miRNA-TF-gene network. The rectangles denote hub genes, ellipses denote miRNA, and hexagons denote TF.

miRNA could function as a regulator in lung injuries. Thus, miRNAs that could interact with the screened hub genes were predicted using CyTargetLinker. And 72 miRNA-target interactions were found in TargetScan, and 207 were found in MicroCosm (Figure 6(a)). The overlap threshold was set to 2 for the analysis, and the results showed that interactions occurred between 18 miRNAs and 5 target genes. TFs play important roles in controlling gene expressions. Therefore, we predicted TFs that could regulate the hub genes using NetworkAnalyst and these TFs were incorporated with the predicted miRNA to construct the miRNA-TF-gene regulatory network (Figure 6(b)). Overall, the regulatory network could help to clarify the roles of miRNA and TFs in the development of VILI.

### 3.6. Analysis of Immune Cell Composition

**Figure 7:**
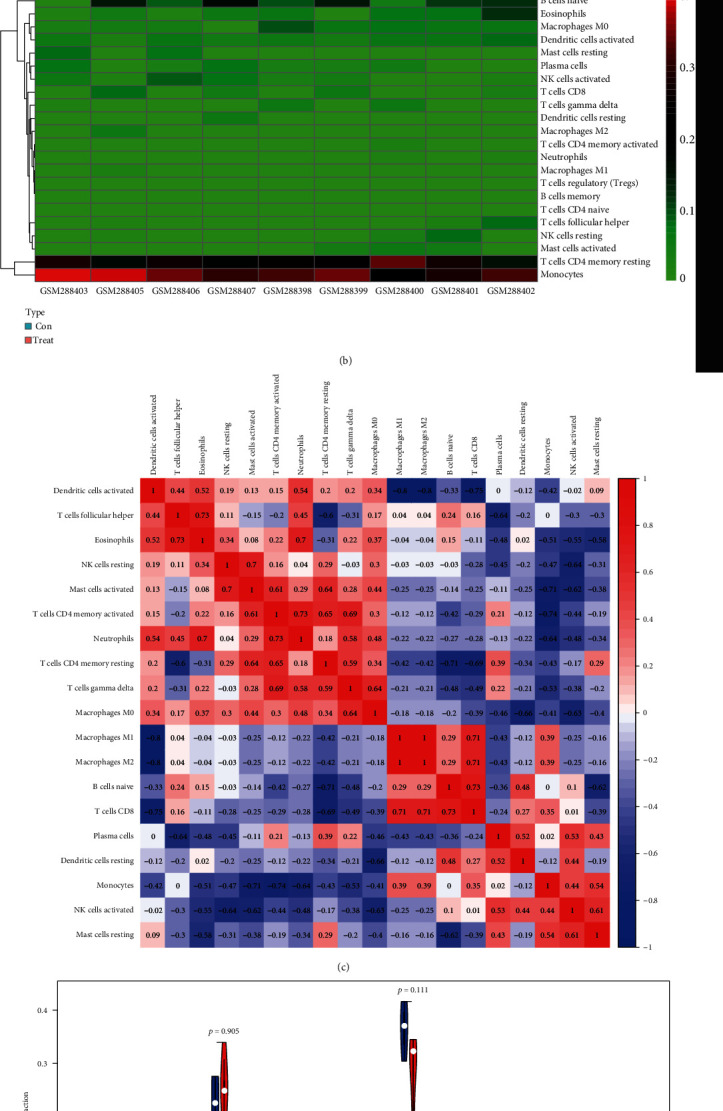
Landscape of immune cell composition in lung samples from the VILI and control groups. (a) Histogram of the immune cell composition of samples from the VILI and control groups. (b) Heat map of immune cell types in lung samples from the VILI and control groups. (c) Correlation matrix of immune cell types in lung samples of the VILI and control groups. (d) Violin plot of immune cell types in lung samples of the VILI and control groups.

To understand the involvement of immune cells in VILI, we used CIBERSORT to detect the contributions of different types of immune cell in the lung tissues of mice in the VILI group. We evaluated immune cell composition in samples from both VILI and control groups (Figure 7(a)). Accordingly, these samples were divided into two main clusters (Figure 7(b)). The three most relevant immune cells included eosinophils and T follicular helper cells, activated neutrophils and memory CD4 T cells, and naive CD8 T cells and B cells, all with an *R* value of 0.49 (Figure 7(c)). It was revealed that the proportion of M0 macrophages and activated mast cells was higher in the VILI group than in the control (Figure 7(d)). The proportion of other immune cells does not show any statistically significant difference between samples from the VILI and control groups. Together, it indicated that immune cells, particularly macrophages, were involved in the early stage of VILI.

### 3.7. Drug Prediction for Hub Genes

DrugBank is a database that provides detailed drug data and information about comprehensive drug targets. To predict possible drugs that may be developed for the treatment of VILI, the DrugBank database was used to identify small molecules that would potentially interact with the hub genes. As shown in Table 5, the most significant molecules included nadroparin, siltuximab, ginseng, donepezil, minocycline, and gallium nitrate.

## 4. Discussion

It has been known that mechanical ventilation could lead to VILI, though the exact pathological process of VILI is still far from clear [6]. Efforts for VILI interventions yield few positive results, and there is no specific treatment for it except preventive measures like low tidal volume ventilation. A better understanding of the underlying molecular mechanism of VILI development, therefore, may help to shed light on the VILI pathogenesis. It is noteworthy that novel tools have been widely used in several health applications. Novel bioinformatics analysis has been applied in analyzing the roles played by mRNA, lncRNA, and miRNA in the VILI development [7, 23, 24]. Xu et al. identified several critical lncRNAs which may help to get a better picture of the VILI pathogenesis [23]. Vaporidi et al. found several differentially regulated miRNAs during the pathological progress of VILI [24]. However, there have been few studies to investigate VILI-associated hub genes using bioinformatics approaches. Besides, miRNA-gene regulatory network has rarely been used in the studies of VILI.

In this study, we acquired the GSE11434 dataset, containing genetic data on the tissues of mice with VILI induced by high-volume ventilation and tissues of control mice, for bioinformatics analysis. A total of 141 DEGs, including 108 upregulated and 33 downregulated genes, were identified. Then, PPI analysis and GSEA were performed to explore their biological significance as regards VILI.

Go enrichment analysis of these DEGs in this study reveals that they are largely enriched in RNA polymerase II promoter and responses to lipopolysaccharide and cAMP. And the roles played by the cAMP signaling pathway include inflammatory response and immune mediator induction [25]. Those results indicate that abnormal inflammatory responses may be involved in the pathological process of VILI.

The PPI network analysis in this study provides some insights for studying the VILI pathogenesis. We constructed a network consisting of DEG-coded proteins. The distribution of degrees, clustering coefficient, distribution of the shortest path, and closeness centrality all demonstrated high connectivity between these proteins. Our network topology analysis confirmed that the network was biologically scale-free. We further applied the MCODE plugin in the PPI network and found that a module consisting of 14 nodes could be the key regulatory network for VILI. The enrichment analysis of this module showed that those genes were largely enriched in inflammatory response, DNA binding, TNF, and JAK-STAT signaling pathways. This finding was consistent with the results in previous studies which revealed that the DNA-binding activity of NF-𝜅B in VILI cases increased [26]. TNF has been reported to be involved in early inflammatory response and stretch-induced pulmonary edema [27, 28]. Subsequently, we used the cytoHubba plugin to further screen out hub genes from the PPI network, which included FOS, STAT3, MYC, ATF3, EGR1, IL-6, and IL-1B. Strikingly, all hub genes were contained in the above-obtained module.

STAT3, an important member of the STAT family, is involved in the transcription activation of the JAK-STAT signaling pathway. Hoegl et al. discovered that IL-22 activates STAT3 signaling and reduces SOCS3 expression, thus alleviating lung injury in VILI cases [29]. In contrast, in our study, the expression of STAT3 is increased in the VILI group, compared with the control. This indicted that STAT3 may have dual effects on the inflammatory process. Wolfson et al. found that STAT3 upregulates HMGB1 expression, thereby exacerbating systemic inflammatory responses in VILI cases [30]. The specific roles of FOS and MYC in VILI have not been systematically studied. FOS can respond to mechanical stimuli and promote immune activation in the lung alveoli [31]. It binds with AP-1 sites to play a role in the proinflammatory signaling pathways in acute lung injuries [32]. Similar to FOS, MYC is transcribed under mechanical stimuli [33].

ATF3 is a transcription factor belonging to the CREB/ATF family. Shan et al. found that ATF3 could protect against lung injury by reducing barrier disruption and inflammatory cell recruitment [34]. Our analysis showed that the ATF3 expression in the VILI group was significantly upregulated compared with the control. Its specific pathophysiological mechanism is unclear and needs to be further studied. EGR1 is a transcription factor in the zinc finger protein family and can be activated by various environmental signals [35]. Copland et al. found that the expression of EGR1 could increase even under low-volume mechanical ventilation, suggesting its role in the upstream regulation [36].

IL-6 is a pleiotropic cytokine that is involved in regulating leukocyte function and apoptosis and thus exhibits proinflammatory and anti-inflammatory effects [37]. Ko et al. reported that NF-*κ*B-IL-6 signaling pathways contribute to the VILI development by inducing inflammation [38]. Experimental lung injury can be attenuated by an IL-1*β* antagonist [39]. This indicates that the inflammatory response may not only be a downstream reaction but also involved in the progression of lung injury. Enrichment analysis of those hub genes identified in this study showed that they were largely enriched in TLR and JAK-STAT inflammatory signaling pathways, a finding that is consistent with the results from previous studies that inflammation is an essential factor in the VILI development.

GSEA could be used to obtain relevant information when large-scale genes were at a small fold change. The GSEA in this study revealed that gene expressions in the VILI samples showed a significant correlation with TLR and JAK-STAT pathways, compared with those in the control samples. In combination with the abovementioned enrichment analysis, we speculate that TLR and JAK-STAT may play vital roles in inflammation observed in VILI cases. Moreover, the antigen presentation pathway was also where enrichment occurred. It could be inferred that the immune system might also be involved in the VILI development. Indeed, a previous study has already raised concerns about involvement of the immune system in VILI [40].

We used CIBERSORT to analyze the immune cell composition in VILI samples and found that the proportion of M0 macrophages and activated mast cells was significantly higher in the VILI group than in the control. Macrophage is known to play a considerable role in the innate immune system. A previous study has found that mechanical ventilation may induce macrophages to switch to the M1 phenotype [41]. That indicates that macrophages could mainly remain as M0 phenotype in the early stage of VILI.

In addition, hub genes were mapped into the DrugBank database to predict small molecular drugs. It is still unclear whether these compounds can contribute therapeutic effects on VILI, so further investigation is required as to whether these molecules can be used to treat VILI in the future.

There are still some limitations in this study. First, changes in gene mutations (such as SNPs), protein expression levels, or cellular metabolism may also play important roles in the occurrence and development of VILI. Limited by a lack of relevant data, we are currently unable to carry out such detailed analysis. Second, our study constructed a PPI network based on transcriptomics data rather than proteomics data. Proteomic analysis of VILI will be conducted in our further studies. Lastly, our study included only a small number of samples, and thus, investigations with a larger sample size shall be further conducted in our future studies.

To summarize, this study systematically analyzed the transcriptomic characteristics of lung tissues from the VILI and control groups to identify DEGs and hub genes. These hub genes, including FOS, STAT3, MYC, ATF3, EGR1, IL-6, and IL-1B, play vital roles in the VILI pathogenesis and thus provide potential therapeutic targets for future VILI treatment.

## 5. Conclusion

In a word, we identified a series of hub genes from the DEGs between the VILI group and the control group, which may provide novel insights into the pathogenesis of VILI and gene targets for its treatment.

## Figures and Tables

**Table 1:** GO analysis of the most significant clustering module in PPI networks.

Category	Term	*p* value
GOTERM_BP_DIRECT	GO:0006351~transcription, DNA-templated	1.19*E* − 04
GOTERM_BP_DIRECT	GO:0045893~positive regulation of transcription, DNA-templated	7.52*E* − 04
GOTERM_BP_DIRECT	GO:0006357~regulation of transcription from RNA polymerase II promoter	7.63*E* − 04
GOTERM_BP_DIRECT	GO:0006954~inflammatory response	0.013427
GOTERM_BP_DIRECT	GO:0043066~negative regulation of apoptotic process	0.014684
GOTERM_BP_DIRECT	GO:0035914~skeletal muscle cell differentiation	0.024883
GOTERM_BP_DIRECT	GO:0051091~positive regulation of sequence-specific DNA binding transcription factor activity	0.048278
GOTERM_BP_DIRECT	GO:0006355~regulation of transcription, DNA-templated	0.048792
GOTERM_CC_DIRECT	GO:0005634~nucleus	3.27*E* − 06
GOTERM_CC_DIRECT	GO:0005654~nucleoplasm	0.039133
GOTERM_MF_DIRECT	GO:0003700~transcription factor activity, sequence-specific DNA binding	1.82*E* − 06
GOTERM_MF_DIRECT	GO:0003677~DNA binding	8.51*E* − 05
GOTERM_MF_DIRECT	GO:0000978~RNA polymerase II core promoter proximal region sequence-specific DNA binding	0.001059
GOTERM_MF_DIRECT	GO:0043565~sequence-specific DNA binding	0.002782
GOTERM_MF_DIRECT	GO:0001077~transcriptional activator activity, RNA polymerase II core promoter proximal region sequence-specific binding	0.009683
GOTERM_MF_DIRECT	GO:0000982~transcription factor activity, RNA polymerase II core promoter proximal region sequence-specific binding	0.014036
GOTERM_MF_DIRECT	GO:0046983~protein dimerization activity	0.015108

**Table 2:** KEGG analysis of the most significant clustering module in PPI networks.

Category	Term	*p* value
KEGG_PATHWAY	ssc04668:TNF signaling pathway	1.84*E* − 08
KEGG_PATHWAY	ssc05166:HTLV-I infection	2.59*E* − 06
KEGG_PATHWAY	ssc05202:transcriptional misregulation in cancer	1.31*E* − 04
KEGG_PATHWAY	ssc05200:pathways in cancer	4.31*E* − 04
KEGG_PATHWAY	ssc04380:osteoclast differentiation	0.001668
KEGG_PATHWAY	ssc04630:JAK-STAT signaling pathway	0.001932
KEGG_PATHWAY	ssc05161:hepatitis B	0.0024
KEGG_PATHWAY	ssc04917:prolactin signaling pathway	0.006385
KEGG_PATHWAY	ssc04931:insulin resistance	0.017635
KEGG_PATHWAY	ssc05169:Epstein-Barr virus infection	0.018235
KEGG_PATHWAY	ssc05152:tuberculosis	0.040023
KEGG_PATHWAY	ssc05168:herpes simplex infection	0.043435

**Table 3:** GO enrichment analysis of hub genes.

Category	Term	*p* value
GOTERM_BP_DIRECT	GO:0035914~skeletal muscle cell differentiation	2.15*E* − 04
GOTERM_BP_DIRECT	GO:0045944~positive regulation of transcription from RNA polymerase II promoter	0.002266
GOTERM_BP_DIRECT	GO:0006351~transcription, DNA-templated	0.002534
GOTERM_BP_DIRECT	GO:0070102~interleukin-6-mediated signaling pathway	0.00354
GOTERM_BP_DIRECT	GO:0043066~negative regulation of apoptotic process	0.006244
GOTERM_BP_DIRECT	GO:0050679~positive regulation of epithelial cell proliferation	0.018091
GOTERM_BP_DIRECT	GO:0006355~regulation of transcription, DNA-templated	0.034263
GOTERM_BP_DIRECT	GO:0042493~response to drug	0.034428
GOTERM_BP_DIRECT	GO:0042593~glucose homeostasis	0.042752
GOTERM_CC_DIRECT	GO:0005654~nucleoplasm	0.016377
GOTERM_CC_DIRECT	GO:0005634~nucleus	0.02582
GOTERM_MF_DIRECT	GO:0005125~cytokine activity	0.001928
GOTERM_MF_DIRECT	GO:0001077~transcriptional activator activity, RNA polymerase II core promoter proximal region sequence-specific binding	0.004063
GOTERM_MF_DIRECT	GO:0000978~RNA polymerase II core promoter proximal region sequence-specific DNA binding	0.008486
GOTERM_MF_DIRECT	GO:0000982~transcription factor activity, RNA polymerase II core promoter proximal region sequence-specific binding	0.011174
GOTERM_MF_DIRECT	GO:0046983~protein dimerization activity	0.017511
GOTERM_MF_DIRECT	GO:0003700~transcription factor activity, sequence-specific DNA binding	0.022809
GOTERM_MF_DIRECT	GO:0008134~transcription factor binding	0.039418
GOTERM_MF_DIRECT	GO:0003677~DNA binding	0.042522

**Table 4:** KEGG enrichment analysis of hub genes.

Category	Term	*p* value
KEGG_PATHWAY	bta05161:hepatitis B	4.74*E* − 06
KEGG_PATHWAY	bta05166:HTLV-I infection	4.92*E* − 05
KEGG_PATHWAY	bta04620:toll-like receptor signaling pathway	8.79*E* − 05
KEGG_PATHWAY	bta05142:Chagas disease (American trypanosomiasis)	1.12*E* − 04
KEGG_PATHWAY	bta05162:measles	2.07*E* − 04
KEGG_PATHWAY	bta04630:JAK-STAT signaling pathway	2.59*E* − 04
KEGG_PATHWAY	bta05020:prion diseases	3.61*E* − 04
KEGG_PATHWAY	bta05168:herpes simplex infection	5.10*E* − 04
KEGG_PATHWAY	bta04623:cytosolic DNA-sensing pathway	0.001314
KEGG_PATHWAY	bta05321:inflammatory bowel disease (IBD)	0.001727
KEGG_PATHWAY	bta05133:pertussis	0.002086
KEGG_PATHWAY	bta05132:Salmonella infection	0.002419
KEGG_PATHWAY	bta05323:rheumatoid arthritis	0.003158
KEGG_PATHWAY	bta04668:TNF signaling pathway	0.004063
KEGG_PATHWAY	bta05200:pathways in cancer	0.00434
KEGG_PATHWAY	bta04380:osteoclast differentiation	0.006194
KEGG_PATHWAY	bta05164:influenza A	0.010163
KEGG_PATHWAY	bta05152:tuberculosis	0.011088
KEGG_PATHWAY	bta04060:cytokine-cytokine receptor interaction	0.015837
KEGG_PATHWAY	bta04010:MAPK signaling pathway	0.021328

**Table 5:** Potential drugs that target hub genes derived from DrugBank.

Gene	Drug	Accession number	Groups	Interaction type
FOS	Nadroparin	DB08813	Approved, investigational	Inhibitor
MYC	Nadroparin	DB08813	Approved, investigational	Inhibitor
IL-6	Siltuximab	DB09036	Approved, investigational	Antagonist
IL-6	Ginseng	DB01404	Approved, investigational, nutraceutical	Antagonist
IL-1B	Donepezil	DB00843	Approved	Inhibitor
IL-1B	Minocycline	DB01017	Approved, investigational	Modulator
IL-1B	Gallium nitrate	DB05260	Approved, investigational	Antagonist

## Data Availability

All data generated or analyzed during this study were included in this accepted article.

## Supplementary Materials

Supplementary Materials Supplemental Table: primers used in qRT-PCR.
