# Supplementary material for: Transcriptome-Wide Gene Expression in a Murine Model of Ventilator-Induced Lung Injury
Source: Dis Markers. 2021 Apr 7;2021:5535890. doi: 10.1155/2021/5535890 (PMC8049808; doi:10.1155/2021/5535890)
Supplement: Supplementary Materials — Supplemental Table: primers used in qRT-PCR. [file 5535890.f1.docx]

Supplemental Table. Primers used in qRT-PCR.

| Gene | Sequence |
| --- | --- |
| GAPDH | Sense: AGGTCGGTGTGAACGGATTTG |
|  | Antisense: TGTAGACCATGTAGTTGAGGTCA |
| ATF3 | Sense: GAGGATTTTGCTAACCTGACACC |
|  | Antisense: TTGACGGTAACTGACTCCAGC |
| FOS | Sense: CGGGTTTCAACGCCGACTA |
|  | Antisense: TTGGCACTAGAGACGGACAGA |
| IL-1β | Sense: GCAACTGTTCCTGAACTCAACT |
|  | Antisense: ATCTTTTGGGGTCCGTCAACT |
| IL6 | Sense: TAGTCCTTCCTACCCCAATTTCC |
|  | Antisense: TTGGTCCTTAGCCACTCCTTC |
| STAT3 | Sense: CAATACCATTGACCTGCCGAT |
|  | Antisense: GAGCGACTCAAACTGCCCT |
| MYC | Sense: ATGCCCCTCAACGTGAACTTC |
|  | Antisense: CGCAACATAGGATGGAGAGCA |
| EGR1 | Sense: ATGCCCCTCAACGTGAACTTC |
|  | Antisense: CGCAACATAGGATGGAGAGCA |
